# Trunk fat volume can be a predictor of postoperative complications after gastrectomy: a retrospective cohort study

**DOI:** 10.1186/s12893-021-01221-3

**Published:** 2021-04-23

**Authors:** Shinichiro Shiomi, Tetsuro Toriumi, Koichi Yagi, Raito Asaoka, Yasuhiro Okumura, Kotaro Wakamatsu, Susumu Aikou, Hiroharu Yamashita, Sachiyo Nomura, Yasuyuki Seto

**Affiliations:** Department of Gastrointestinal Surgery, Graduate School of Medicine, University of Tokyo, 7-3-1 Hongo, Bunkyo-ku, Tokyo, 113-8655 Japan

**Keywords:** Gastrectomy, Obesity, Intra-abdominal fat, Postoperative complication

## Abstract

**Background:**

Obesity can affect postoperative outcomes of gastrectomy. Visceral fat area is superior to body mass index in predicting postoperative complications. However, visceral fat area measurement is time-consuming and is not optimum for clinical use. Meanwhile, trunk fat volume (TFV) can be easily measured via bioelectrical impedance analysis. Hence, the current study aimed to determine the ability of TFV to predict the occurrence of complications after gastrectomy.

**Methods:**

We retrospectively reviewed patients who underwent curative gastrectomy for gastric cancer between November 2016 and November 2019. The trunk fat volume-to-the ideal amount (%TFV) ratio was obtained using InBody 770 before surgery. The patients were classified into the obese and nonobese groups according to %TFV (TFV-H group, ≥ 150%; TFV-L group, < 150%) and body mass index (BMI-H group, ≥ 25 kg/m^2^; BMI-L group, < 25 kg/m^2^). We compared the short-term postoperative outcomes (e.g., operative time, blood loss volume, number of resected lymph nodes, and duration of hospital stay) between the obese and nonobese patients. Risk factors for complications were assessed using logistic regression analysis.

**Results:**

In total, 232 patients were included in this study. The TFV-H and BMI-H groups had a significantly longer operative time than the TFV-L (*p* = 0.022) and BMI-L groups (*p* = 0.006). Moreover, the TFV-H group had a significantly higher complication rate (*p* = 0.004) and a lower number of resected lymph nodes (*p* < 0.001) than the TFV-L group. In the univariate analysis, %TFV ≥ 150, total or proximal gastrectomy, and open gastrectomy were found to be potentially associated with higher complication rates (*p* < 0.1 for all). Moreover, the multivariate analysis revealed that %TFV ≥ 150 (OR: 2.73; 95% CI: 1.37–5.46; *p* = 0.005) and total or proximal gastrectomy (OR: 3.57; 95% CI: 1.79–7.12; *p* < 0.001) were independently correlated with postoperative morbidity.

**Conclusions:**

%TFV independently affected postoperative complications. Hence, it may be a useful parameter for the evaluation of obesity and a predictor of complications after gastrectomy.

## Background

Gastric cancer is the sixth most common cancer worldwide [1], and gastrectomy has been considered essential for its curative treatment. Postoperative complications adversely affect the long-term survival of patients with gastric cancer. Therefore, the risk factors of postoperative complications should be identified and postoperative complication rates reduced [2].

Excessive intra-abdominal fat tissue poses difficulties during surgery. Obesity is associated with short-term surgical outcomes, including complications after gastrectomy [3, 4], and is evaluated using several methods. Body mass index (BMI) is the most widely used tool for obesity assessment because it is easy to use, and usually, individuals with BMI > 25 kg/m^2^ are considered obese in Japan [5]. Visceral fat area (VFA), which refers to intra-abdominal fat, is another index used to evaluate obesity. It is measured at the level of the umbilicus on single-slice computed tomography (CT) scan. Recently, several studies have reported that BMI cannot accurately reflect perioperative outcomes, and VFA is more accurate in predicting short-term postoperative outcomes [3, 4, 6–8]. However, measuring VFA is sometimes difficult at some institutions owing to various reasons, including labor shortages and technical limitations.

Trunk fat volume (TFV), which reflects the fat mass of the body trunk, is another parameter that can be used to evaluate obesity. TFV can be easily measured using bioelectrical impedance analysis (BIA). BIA is a simple method to measure body compositions, including fat mass; this method is increasingly used worldwide because it is noninvasive, cost-effective, and simple to use [9]. Recent studies have shown that body composition measurements can help in evaluating nutrition status after surgery and in predicting surgical outcomes [10]. However, to the best of our knowledge, the relationship between TFV and postoperative outcomes has not been reported in previous studies. Hence, this study aimed to identify the efficacy of TFV in predicting outcomes after gastrectomy.

## Methods

### Patients

This was a single institutional retrospective cohort study conducted at the University of Tokyo Hospital. In total, 278 patients underwent gastrectomy for gastric cancer from November 2016 to November 2019. At our institution, preoperative examinations included upper gastrointestinal endoscopy with biopsies, CT scans, and laboratory tests. Moreover, we routinely measure body composition preoperatively using BIA with written consent. Clinical cancer stage was determined according to the Japanese Classification of Gastric Carcinoma (15th edition) [11]. Treatment strategies were mainly based on the Japanese gastric cancer treatment guidelines (4 and 5th editions) [12, 13]. Patients who could not undergo curative resection and those who received neoadjuvant chemotherapy were excluded. Study procedures were performed in accordance with the Declaration of Helsinki. The ethics committee of the Faculty of Medicine at the University of Tokyo approved this study and waived the requirement for informed consent as anonymized data were used (approval number: 3962).

### Definition of obesity assessed using BMI and TFV

The patients were divided into obese and nonobese groups according to both BMI and TFV. Patients with BMI ≥ 25 kg/m^2^ were classified under the BMI high (BMI-H) group and patients with BMI < 25 kg/m^2^ under the BMI low (BMI-L) group. We obtained the TFV and body fat mass (BFM) using BIA with InBody 770® (InBody Co., Ltd., Seoul, Korea) one or two days before surgery. This tool uses direct segmental multifrequency BIA. It introduces alternating currents into the body and measures impedance, which comprises resistance and reactance. Moreover, it uses eight electrodes and individually measures the impedance of each body part (e.g., the trunk, right and left arms, and right and left legs) at six different frequencies to evaluate body compositions [14, 15]. The accuracy of BIA has been evaluated in several studies, and it is correlated with standard body composition parameters obtained using different modalities, such as dual-energy X-ray absorptiometry, CT scan, and air displacement plethysmography [9, 14, 16, 17]. We classified patients according to TFV in the following manner. First, we calculated the TFV-to-BFM ratios. The median in each sex was defined as an ideal distribution of trunk body fat (% in men and % in women, respectively). Second, we calculated the ideal BFM by multiplying the ideal body weight (height × height × 22 for men and height × height × 21 for women [18]) with the ideal percentage of body fat (15% for men and 23% for women [19]). Third, we defined the product by multiplying these two components as the ideal TFV. Finally, we calculated the TFV-to-the ideal TFV (%TFV) ratio of each patient. The median TFV-to-BFM ratio of men and women were 0.50 and 0.47, respectively. Following these calculations, we derived the following equations according to sex.

$${\text{Men}}:{\text{\% TFV}} = \frac{{{\text{TFV}}}}{{({\text{height}}^{2} \times 21) \times 0.15 \times 0.50}}$$

$${\text{Women}}:{\text{\% TFV}} = \frac{{{\text{TFV}}}}{{({\text{height}}^{2} \times 22) \times 0.23 \times 0.47}}$$

Since no study has reported the determination of obesity using TFV, we selected the threshold of obesity for TFV according to body fat percentage. The cutoff values of body fat percentage for obesity were reported to be 23.67% for men and 32.88% for women [20]. The ratios of the cutoff values to the aforementioned ideal values [19] were 158% and 143%, respectively. Based on these results, patients with %TFV ≥ 150% and %TFV < 150% were classified under the high TFV (TFV-H) and low TFV (TFV-L) groups, respectively.

### Surgical procedures

As one of the roles of our institution is surgeon education, 25 surgeons including trainees performed gastrectomy during the study period. However, all surgical procedures were supervised by six experienced surgeons certified by the Japan Society of Gastrointestinal Surgery. We performed distal gastrectomy, total gastrectomy, proximal gastrectomy, or pylorus-preserving gastrectomy with radical lymphadenectomy as per the treatment guidelines of the Japanese Gastric Cancer Association [12, 13]. Laparoscopic gastrectomy was used in the preoperative diagnosis of T1-2N0 tumors. Patients who underwent distal or total gastrectomy had Roux-en-Y reconstruction. Esophagogastric anastomosis, jejunal interposition anastomosis, or side overlap esophagogastrostomy that is based on the study of Yamashita [21] was conducted for the reconstruction of proximal gastrectomy. A gastro-gastro anastomosis was established for the reconstruction of pylorus-preserving gastrectomy.

### Evaluation of short-term postoperative outcomes

We have compared the short-term postoperative outcomes, including operative time, volume of blood loss, number of resected lymph nodes, and duration of hospital stay between the obese and nonobese patients. Moreover, the risk factors for complications were assessed. The Clavien–Dindo classification system [22] was used to assess postoperative morbidity. Complications greater than grade 2 were considered as clinically significant and those greater than grade 3a as severe.

### Statistical analysis

The Pearson’s chi-square test or Fisher’s exact test was used in the univariate analysis. Meanwhile, logistic regression was utilized in the multivariate analysis, which was performed using factors with p values < 0.1 in the univariate analysis. The student’s *t*-test or Wilcoxon signed-rank test was utilized for continuous value. Further, p values < 0.05 were considered statistically significant. JMP® 15 (SAS Institute Inc., Cavy, NC, the USA) was utilized for all statistical analyses.

## Results

### Characteristics of the patients

**Fig. 1:**
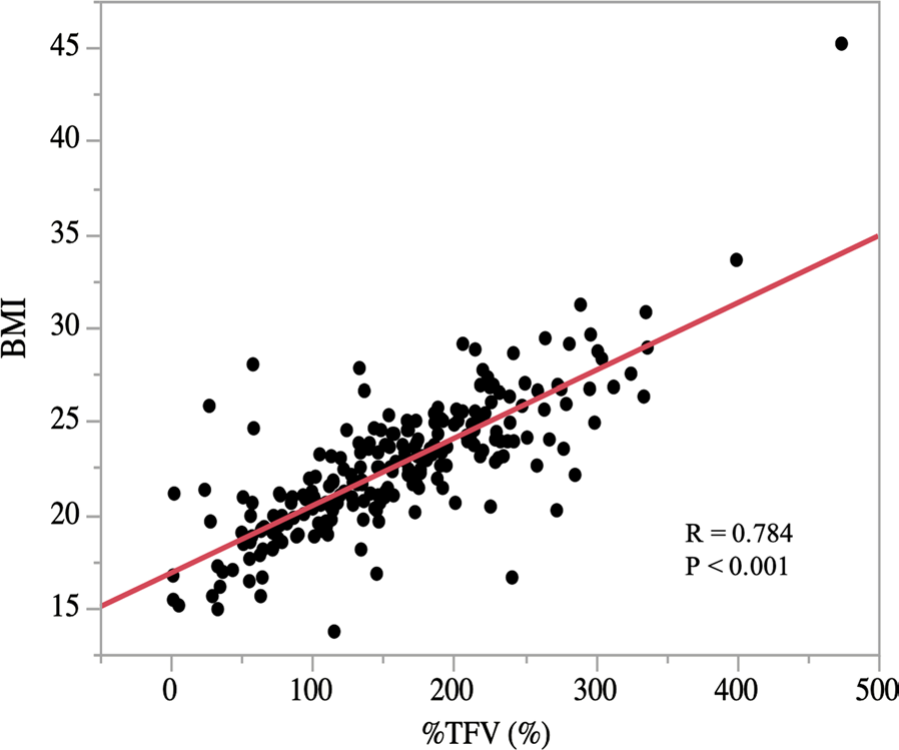
Trunk fat volume-to-the ideal amount ratio (%TFV) correlated with body mass index (r = 0.784)

Of 278 patients, 14 who underwent R1 or R2 resection, and 32 who received neoadjuvant chemotherapy were excluded. Finally, 232 patients were included in our study. The characteristics of the patients are shown in Table 1. In both criteria, obese patients underwent significantly more open gastrectomy and had more severe comorbidities. Besides, the TFV-H group had a higher proportion of male patients, early-stage disease, and D1 + dissection, which were not evident in the BMI group. Other properties were comparable between the two groups. A significant correlation was observed between %TFV and BMI (r = 0.784, *p* < 0.001) (Fig. 1).

**Table 1:** Characteristics of the patients

		TFV-H group (n = 120)	TFV-L group (n = 112)	*p* value	BMI-H group (n = 50)	BMI-L group (n = 182)	*p* value
Sex							
Male		92 (76.7%)	67 (59.8%)	0.006	35 (70.0%)	124 (68.1%)	0.865
Female		28 (23.3%)	45 (40.2%)		15 (30.0%)	58 (31.9%)	
Age (years), (mean ± SD)		70.1 ± 10.0	68.9 ± 12.8	0.411	66.8 ± 11.8	70.3 ± 11.3	0.056
%TFV (%), (mean ± SD)		219.7 ± 54.9	94.0 ± 38.5	< 0.001	289.1 ± 68.0	152.1 ± 69.1	< 0.001
BMI (kg/m^2^), (mean ± SD)		24.7 ± 3.2	20.3 ± 2.6	< 0.001	27.4 ± 3.1	21.2 ± 2.4	< 0.001
Clinical stage							
I		102 (85.0%)	68 (60.7%)	< 0.001	42 (84.0%)	123 (70.3%)	0.204
II A		0 (0.0%)	7 (6.3%)		0 (0.0%)	7 (3.9%)	
II B		12 (10.0%)	25 (22.3%)		5 (10.0%)	32 (17.6%)	
III		6 (5.0%)	12 (10.7%)		4 (8.0%)	15 (8.2%)	
Open gastrectomy		24 (20.0%)	45 (40.2%)	< 0.001	7 (14.0%)	62 (34.1%)	0.005
Total/proximal gastrectomy		33 (27.5%)	22 (19.6%)	0.160	10 (20.0%)	45 (24.7%)	0.487
LD							
≤ D1 +		83 (69.2%)	59 (52.7%)	0.010	34 (68.1%)	108 (59.3%)	0.327
≥ D2		37 (30.8%)	53 (47.3%)		16 (32.0%)	74 (40.7%)	
CCI							
≥ 3		12 (10.0%)	4 (3.6%)	0.054	7 (14.0%)	9 (5.0%)	0.025
≤ 2		108 (90.0%)	108 (96.4%)		43 (86.0%)	173 (95.0%)	
Operative history		28 (23.3%)	35 (31.3%)	0.176	9 (18.0%)	54 (29.7%)	0.111

*TFV* trunk fat volume, *SD* standard deviation, *BMI* body mass index, *LD* lymph node dissection, *CCI* Charlson comorbidity index

### Correlation between obesity and short-term outcomes

Table 2 shows the short-term outcomes between the obese and nonobese groups. The TFV-H group required a significantly longer operative time than the TFV-L group (305.1 ± 67.5 vs 284.9 ± 66.0 min, *p* = 0.022). Moreover, the TFV-H group had a lower number of resected lymph nodes (33 [interquartile range {IQR} 25–43] vs 42 [IQR 32–52.75], *p* < 0.001) than the TFV-L group. Although the BMI-H group had a significantly longer operative time than the BMI-L group (321.9 ± 78.5 vs 288.1 ± 62.2 min, *p* = 0.006), the number of resected lymph nodes did not differ significantly between the two groups. The volume of blood loss and duration of hospital stay were comparable between the two groups in both criteria.

**Table 2:** Short-term surgical outcomes between obese and nonobese patients

	TFV-H group (n = 120)	TFV-L group (n = 112)	*p* value	BMI-H group (n = 50)	BMI-L group (n = 182)	*p* value
Operative time (min), (mean ± SD)	305.1 ± 67.5	284.9 ± 66.0	0.022	321.9 ± 78.5	288.1 ± 62.2	0.006
Volume of blood loss^1^ (mL), (median [IQR])	100 (20–218.75)	85 (10–216.25)	0.279	117.5 (27.5–236.3)	75 (10–207.5)	0.148
Number of resected LN^1^ (median [IQR])	33 (25–43)	42 (32–52.75)	< 0.001	34 (26–43)	38 (29–48)	0.112
Duration of hospital stay^1^ (day), (median [IQR])	9 (8–12)	10 (9–13)	0.135	10 (9–13)	10 (9–12)	0.358

^1^Variables are indicated by median

*TFV* trunk fat volume, *SD* standard deviation, *BMI* body mass index, *IQR* interquartile range, *LN* lymph node

### Risk factors of postoperative complications

We evaluated the risk factors of postoperative complications. The overall complication rate was 24.6%. Table 3 shows the results of the univariate and multivariate analyses. In the univariate analysis, %TFV ≥ 150 (32.5 vs 16.1%, *p* = 0.004), total or proximal gastrectomy (47.3 vs 17.5%, *p* < 0.001), and open surgery (31.9 vs 21.5%, *p* = 0.092) were found to be factors potentially associated with higher complication rates with *p* values < 0.1. The multivariate analysis revealed that %TFV ≥ 150 (odds ratio [OR]: 2.73; 95% confidence interval [CI]: 1.37–5.46; *p* = 0.005) and total or proximal gastrectomy (OR: 3.57; 95% CI: 1.79–7.12; *p* < 0.001) were independently associated with postoperative morbidity.

**Table 3:** Risk factors of postoperative complications

		Complications ( +) (n = 57)	Complications (−) (n = 175)	Univariate analysis	Multivariate analysis	
					Odds ratio (95% CI)	*p* value
Sex						
Male (159)		43 (27.0%)	116 (73.0%)	0.196		
Female (73)		14 (19.2%)	59 (80.8%)			
Age (years)						
≥ 65(172)		45 (26.1%)	127 (73.9%)	0.340		
< 65(60)		12 (20.0%)	48 (80.0%)			
BMI (kg/m^2^)						
≥ 25(50)		13 (26.0%)	37 (74.0%)	0.791		
< 25(182)		44 (24.2%)	141 (77.5%)			
%TFV						
≥ 150 (120)		39 (32.5%)	81 (67.5%)	0.004	2.73 (1.37–5.46)	0.005
< 150 (112)		18 (16.1%)	94 (83.9%)			
cStage						
I (170)		39 (22.9%)	131 (77.1%)	0.340		
II A/II B/III (62)		18 (29.0%)	44 (71.0%)			
Surgery						
OP (69)		22 (31.9%)	47 (68.1%)	0.092	1.73 (0.84–3.57)	0.137
LAP (163)		35 (21.5%)	128 (78.5%)			
Procedure						
TG/PG (55)		26 (47.3%)	29 (52.7%)	< 0.001	3.57 (1.79–7.12)	< 0.001
DG/PPG (177)		31 (17.5%)	146 (82.5%)			
LD						
≤ D1 + (142)		33 (23.2%)	109 (76.8%)	0.555		
≥ D2 (90)		24 (26.7%)	66 (73.3%)			
CCI						
≥ 3 (16)		6 (37.5%)	10 (62.5%)	0.213		
< 2 (216)		51 (23.6%)	165 (76.4%)			
Operative history						
Yes (63)		12 (19.1%)	51 (80.9%)	0.233		
No (169)		45 (26.6%)	124 (73.4%)			

*CI* confidence interval, *TFV* trunk fat volume, *BMI* body mass index, *LD* lymph node dissection, *CCI* Charlson comorbidity index,

*OP* open, *LAP* laparoscopic, *TG* total gastrectomy, *DG* distal gastrectomy, *PPG* pylorus-preserving gastrectomy, *PG* proximal gastrectomy

### Details of postoperative complications

The details of postoperative complications are presented in Table 4. Severe complications were also more common in the TFV-H group than in the TFV-L group although the difference was not significant (10.8 vs 4.5%, *p* = 0.070). In terms of complications, anastomotic leakage, pancreatic fistula, and pneumonia were more common in the TFV-H group than in the TFV-L group. However, only the occurrence of pancreatic fistula was statistically significant (5.8 vs 0.9%, *p* = 0.039). Meanwhile, the rate of each complication in the BMI-H group was comparable with that in the BMI-L group.

**Table 4:** Details of postoperative complications after gastrectomy

	TFV-H group (n = 120)	TFV-L group (n = 112)	*p* value	BMI-H group (n = 50)	BMI-L group (n = 182)	*p* value
Complications (Clavien–Dindo classification score ≥ 2)	39 (32.5%)	18 (16.1%)	0.004	13 (26.0%)	44 (24.1%)	0.791
Severe complications (Clavien–Dindo classification score ≥ 3a)	13 (10.8%)	5 (4.5%)	0.070	4 (8.0%)	14 (7.7%)	0.943
Pneumonia	6 (5.0%)	1 (0.9%)	0.068	3 (6.0%)	4 (2.2%)	0.164
Anastomotic leakage	7 (5.8%)	3 (2.7%)	0.237	2 (4.0%)	8 (4.4%)	0.902
Pancreatic fistula	7 (5.8%)	1 (0.9%)	0.039	3 (6.0%)	5 (2.8%)	0.264
Incisional SSI	3 (2.5%)	6 (4.5%)	0.413	1 (2.0%)	7 (3.9%)	0.526
Anastomotic stenosis	3 (2.5%)	0 (0.0%)	0.092	0 (0.0%)	3 (1.7%)	0.361
Bleeding	1 (0.8%)	1 (0.9%)	0.961	0 (0.0%)	2 (1.1%)	0.457
Bowel obstruction	2 (1.7%)	0 (0.0%)	0.170	1 (2.0%)	1 (0.6%)	0.326
FUO	2 (1.7%)	1 (0.9%)	0.602	1 (2.0%)	2 (1.1%)	0.617
Delayed gastric ejection	3 (2.5%)	3 (2.7%)	0.932	0 (0.0%)	6 (3.3%)	0.193
Others	8 (6.7%)	3 (2.7%)	0.153	2 (4.0%)	9 (5.0%)	0.781

*TFV* trunk fat volume, *BMI* body mass index, *SSI* surgical site infection, *FUO* fever of unknown origin

## Discussion

This study revealed that obesity evaluated using %TFV and total or proximal gastrectomy was independently associated with postoperative complications. The %TFV may be a better parameter than BMI for the prediction of postoperative complications such as pancreatic fistula. These findings indicate that TFV may help to determine the necessity of drain management or frequent following up of blood tests and X-ray examination in the postoperative period and enable the early detection of each complication.

Excessive visceral fat poses difficulties during surgery, and obesity is associated with unfavorable surgical outcomes, including longer operative time, higher postoperative complication rates, lower number of resected lymph nodes, and prolonged hospital stay [4, 8, 23, 24]. The results of this study are consistent with the results of previous reports. However, there were some differences in the background of patients included in this study. Compared with patients in the nonobese group, a higher proportion of patients in the obese group underwent laparoscopic gastrectomy. Since laparoscopic gastrectomy reportedly requires more time than laparotomy [25], the difference in operative time may be greatly affected by differences in background and body fat; thus, this data should be taken into consideration. Moreover, the proportion of female patients in the TFV-H group was significantly lower than that in the TFV-L group, which was not observed in the groups classified according to BMI. Theoretically, fat mass is more likely to accumulate in the visceral area in men than in women [6], and gastrectomy is mainly affected by visceral fat [26]. Hence, the complication rate might be lower in females, and the deviation in female distribution may affect the discordance in morbidity risk between %TFV and BMI. However, sex was not a significant risk factor affecting complications in the multivariate analysis. In addition, a higher complication rate was observed in TFV-H patients than that in TFV-L patients for both males (34.8% vs 16.4%) and females (28.6% vs 13.3%), which was not the case in the BMI-H and L groups (data not shown).

In this study, pancreatic fistula and anastomotic leakage were more common in the obese group classified according to TFV, and this result was in accordance with that of a previous study [27]. Previous studies have reported the possible causes of poor outcomes among obese patients. High visceral fat may be associated with the misrecognition of anatomy and technical difficulty in achieving a good view of the surgical field. Occasionally, this causes excessive countertraction and overcompression in the pancreas during lymph node dissection [8, 26–30]. These factors result in tissue trauma, increased volume of blood loss, prolonged operative time, and pancreatic fistula. Excessive tension on the anastomosis site due to thick and heavy mesenteric fat may be a risk factor for anastomotic leakage [23, 29]. In this study, pneumonia was more frequently observed in the obese group, which was consistent with the result of a previous study [31]. Obesity is associated with decreased total lung capacity attributed to high intra-abdominal pressure or excessive subcutaneous fat around the thorax [32]. Difficulty in clearing airway secretions and delayed ambulation may be contributory factors for pulmonary complications, such as atelectasis and pneumonia. However, the number of patients who had each complication was extremely low in this study, and only the occurrence of pancreatic fistula showed a significant difference. Thus, further investigations should be conducted to accurately identify the occurrence of complications after surgery.

BMI is the most commonly used parameter for the evaluation of obesity. Several studies have shown that BMI was not a predictive factor of postoperative complications [3, 6, 8]. This is believed to be due to the simplicity of BMI as BMI is calculated using only height and weight and does not directly reflect fat volume [7, 8, 26]. On the contrary, some studies have demonstrated that higher BMI was associated with worse surgical outcomes [24, 27, 28], and thus, whether obesity determined using BMI can be used to predict operative risks remains controversial. Moreover, further investigations on the cutoff value are warranted. Although BMI of 25 is a widely accepted definition of obesity in Asia and most studies have used this cutoff value [3, 4, 6–8, 24, 27, 28], in several studies, including this study, there was a deviation in distribution between obese and nonobese patients [3, 7, 8]. This deviation might have affected the results and indicates that the cutoff value of 25 used to define obesity may be inappropriate for predicting the risk of postoperative complications. Hence, further studies are required to resolve this issue. In this study, we used %TFV obtained using BIA, which has been increasingly used in recent studies [14, 15]. Theoretically, %TFV can only evaluate trunk fat mass, whereas BMI assesses whole body elements including muscles and extremities. Therefore, the use of %TFV may be more suitable for the prediction of surgical risk. Our results were consistent with this theory. Another parameter that is used to evaluate obesity is VFA, which may directly reflect intra-abdominal fat and enable a more accurate prediction of postoperative complications compared with BMI [3, 8, 29, 30]. However, the measurement of VFA is difficult at our institution due to technical limitations involving CT image analysis. Therefore, VFA is not always suitable for clinical use, and other parameters are preferred at institutions with such limitations. %TFV may be inferior to VFA because it cannot distinguish subcutaneous fat from visceral fat. However, both parameters can provide a more accurate assessment of surgical risk than BMI. Hence, %TFV may be an alternative to VFA in institutions lacking modalities for routine VFA assessment.

Preoperative exercise intervention has been reported to be beneficial, especially in obese patients [33]. When we conduct preoperative interventions, there is a concern about tumor progression, particularly in patients with advanced cancer. However, preoperative wait time up to 90 days has been reported to not affect survival even in cStage II/III gastric cancer patients [34]. Hence, obese patients might have the benefits of fewer complications by preoperative exercise. In this study, %TFV ≥ 150 was an independent risk factor for postoperative complications, and individuals with %TFV ≥ 150 might be good candidates for preoperative intervention, although further study is warranted for a firm conclusion on the same.

The current study had several limitations. First, this was a retrospective, single-center study. Hence, it was susceptible to selection and cognitive bias. Hence, a larger multicenter study should be performed to obtain a firm conclusion. Second, different surgeons performed gastrectomy during the study period. Hence, the differences in the ability of the surgeons might have affected the results. Although skilled surgeons supervised trainee surgeons who performed gastrectomy, the effect might still be significant. Third, the ideal TFV was derived from the median TFV-to-BFM ratio, which was obtained from data in this study because no previous studies have discussed the ideal distribution of body fat. However, this value should be determined based on the general population. Finally, given the difference in fat distribution between men and women, the correlation between %TFV and postoperative complications may not be the same in both sexes, and the validity of using a similar %TFV threshold in both men and women is yet to be confirmed. Further studies should be conducted to identify the ideal threshold for each sex.

## Conclusions

Obesity evaluated using %TFV, but not BMI, is an independent risk factor of postoperative complications. %TFV can be an alternative parameter for predicting postoperative complications.

## Data Availability

All data generated or analyzed during this study are included in this published article.

## Abbreviations

BFM: Body fat mass
BIA: Bioelectrical impedance analysis
BMI: Body mass index
CT: Computed tomography
TFV: Trunk fat volume
VFA: Visceral fat area
